# Biobjective Optimization Algorithms Using Neumann Series Expansion for Engineering Design

**DOI:** 10.1155/2018/7071647

**Published:** 2018-12-19

**Authors:** Huan Guo, Yoshino Tatsuo, Lulu Fan, Ao Ding, Tianshuang Xu, Genyuan Xing

**Affiliations:** ^1^School of Mechanical Science and Engineering, Jilin University, Changchun, China; ^2^Aviation University of Air Force, Changchun, China; ^3^Tianjin Aerisafety Science and Technology Co. Ltd., Tianjin, China

## Abstract

In this paper, two novel algorithms are designed for solving biobjective optimization engineering problems. In order to obtain the optimal solutions of the biobjective optimization problems in a fast and accurate manner, the algorithms, which have combined Newton's method with Neumann series expansion as well as the weighted sum method, are applied to deal with two objectives, and the Pareto optimal front is achieved through adjusting weighted factors. Theoretical analysis and numerical examples demonstrate the validity and effectiveness of the proposed algorithms. Moreover, an effective biobjective optimization strategy, which is based upon the two algorithms and the surrogate model method, is developed for engineering problems. The effectiveness of the optimization strategy is proved by its application to the optimal design of the dummy head structure in the car crash experiments.

## 1. Introduction

It is very important to research on the multiobjective optimization problems in the engineering designs. For example, the economist fuel, the maximum carrying capacity, and the lightest weight need to be considered at the same time in the design of aircraft and spacecraft [1]; the strong rigidity, lightweight, and low-order modes also need to be considered commonly in the thin-walled beam section optimization problem of the automobile body structure design [2]. According to the investigation on the dummy head under automobile impact condition, the peak of synthetic acceleration of frontal and lateral drop is the main indicators of mechanical characteristics of dummy head [3]. A bilevel optimization was carried out for the cross-sectional shape of a thin-walled car body frame constrained with static and dynamic stiffness [4]. The common coin of these engineering problems is complex mechanical structure and too much design variables always lead to the intricate solving procedure and large amount of computation with the purpose of multiple objectives meeting the best simultaneously. However, in most cases, the change of one target may cause an influence on the other ones. It is almost impossible to get a solution which can make each objective function reach the optimal value [5]. Therefore, the importance of carrying out the research of multiobjective optimization is of significance to engineering itself especially.

In most cases, an optimal solution which meets all objectives at the same time in a multiobjective problem does not exist. Thus, the key to describe an optimization problem is establishing a scientific and reasonable standard. However, it is also an effective and acceptable way to keep all object values on a relatively better level in the case that the optimal solutions cannot be obtained simultaneously. So, designers can choose one from several groups of the relatively better designs to guide decisions based on engineering background knowledge. The concept of Pareto optimal solution in a multiobjective optimization problem is an objective description which can take into account of every object thoroughly, so that optimization schemes can be calculated by designers in the circumstances of keeping the whole optimization level from dropping [6].

The idea of solving numerical multiobjective optimization problem is a scalarization process, which uses a suitable scalar (single objective) optimization problem instead of the vector (multiobjective) optimization problem [7]. The commonly used algorithms are provided for scalarization process such as minimax method [8], constraint method [9], and usual weighted sum method [10].

The proposed minimax method is a classical multiobjective optimization algorithm [8]. By proving that the set of Pareto optimal solutions coincides with the set of stationary points, it is a parameter-free optimization method for computing a point satisfying a certain first-order necessary condition in multiobjective optimization. It borrows the idea of Newton's method for single-objective optimization and with respect to the authors' theoretical results obtained; Newton's method for multiobjective optimization behaves exactly as its counterpart for single-criterion optimization: it is fairly robust with respect to the dimension of the problem and the starting point chosen, the rate of convergence is at least superlinear, and it is quadratic if the second derivatives are Lipschitz-continuous. But the authors did not discuss the adaptation of the approach they proposed for constrained multiobjective problems. Quasi-Newton's method for solving multiobjective was proposed by Qu et al. [11] and Povalej [12]. By using the well-known BFGS method and the idea of [8], the authors had proven that quasi-Newton's method for multiobjective optimization converges superlinearly to the solution of the given problem, if all functions involved are twice continuously differentiable and strongly convex. The advantage of this method, compared to Newton's approach, is that the approximation of Hessian matrices is usually reasonably faster than their actual evaluation. This difference is especially noticeable when the dimension of the problem rises. The adaptation of this approach to constrained multiobjective optimization is not considered too.

The representative constraint method is *ε*-constraint method [9]; it retains the objective function which designers most prefer, as an objective function of single-objective optimization function, turning other objective functions into constraints by adding a restriction domain *ε*_*i*_ [13]. This algorithm has high efficiency and produces Pareto solutions which have a relatively broad range and does not need to make a priority of getting each objective function in grading (to determine the weight) [6]. However, *ε*-constraint method cannot guarantee that the result is a Pareto optimal solution; selecting an appropriate constraint value often requires some prior knowledge and has a low calculating efficiency when the number of objective function increases. The main drawbacks of these common methods are the limitations on calculation and dissatisfactions with the quality of Pareto optimal solutions [14].

The weighted sum method has been widely used because of its simplicity and high computational efficiency [15]. The early usual weighted sum method transforms multiple objectives into an aggregated objective function by multiplying each objective function by a weighted factor and adding them up. But it has two drawbacks: difficulty to obtain Pareto optimal solutions uniformly and failure to solve nonconvex problems [16–18].

Many methods for solving nonconvex optimization problems have been proposed over the decades. Typical one is the normal-boundary intersection method (NBIM) [19]. It approaches a group of Pareto optimal solutions through geometric intuition parametric method and gives an accurate pattern of Pareto front. NBIM can not only obtain the Pareto optimal solutions in nonconvex regions but also the solutions are uniformly distributed. However, there are still serious defects, for example, non-Pareto optimal solutions (dominated solutions) are also obtained which must be filtered out. The adaptive weighted sum method (AWSM) is presented for the biobjective optimization problems [20], by adding inequality constraints based on traditional weighted sum methods and redefining feasible regions of optimization problems. So, the solve area is extended, and the optimal solutions are iterated automatically.

There are many biobjective optimization problems in engineering applications. The increase of each objective value will immediately cause an influence on another one. For example, energy-absorbing and impact force are a typical pair of contradictory optimized objectives in bumper-crash box design, which needs to make the maximum of impact force decrease while maximize energy absorption to the peak value. But in the practical engineering, with the rising of energy absorption, the impact force of the crash box will be even greater. So, the biobjective optimization is of great significance in engineering.

In this paper, two new algorithms for biobjective optimization problem are presented. One is Newton Neumann Series Expansion Algorithm (NNSEA) for unconstrained problem and Newton Neumann Series Expansion Frisch Algorithm (NNSEFA) for constrained problems. Two examples are given to demonstrate the valid and effectiveness of the algorithms, respectively. Finally, two algorithms are applied to the optimization problem in a dummy head design, and some good results are obtained. The following sections will discuss them in detail.

## 2. Newton Weighted Sum Algorithm for Unconstrained Multiobjective Optimization

### 2.1. Definition and Some Theories of the Multiobjective Optimization Problems

In order to accurately describe the concept of Pareto optimal solution, some definitions and symbols of multiobjective optimization will be presented first.

In this paper, denote by *N*_+_ the positive integer set, by *R* the real number set, by *R*^*n*^ the *n*-dimensional real vector space, and by *R*^*n*×*n*^ the linear space which is composed of *n*-order real matrix. The Euclidean norm in *R*^*n*^ will be denoted by ‖·‖, and we will use the same notation ‖·‖ to describe the induced operator norms on the corresponding matrix spaces. **x** = (*x*_1_, *x*_2_,…,*x*_*n*_)^*T*^ ∈ *R*^*n*^ is a vector of design variables. *F*(**x**) = (*f*_1_(**x**), *f*_2_(**x**),…,*f*_*l*_(**x**))^*T*^ ∈ *R*^*l*^ is the vector-valued objective function which components *f*_*j*_(**x**) = *f*_*j*_(*x*_1_, *x*_2_, ⋯, *x*_*n*_) are *n*-variable real functions for all *j* = 1, 2,…, *l*.

A general multiobjective optimization problem can be defined as follows:

(1) $$\begin{array}{c} \left\{\begin{array}{c} \min\;F\left(x\right),\\ s.t.\;x\in U\subset R^{n}, \end{array}\right. \end{array}$$

where *F*(**x**) is called the objective vector-valued function and *U* is the feasible region of (1). *U* can be described by

(2) $$\begin{array}{c} U=\left\{x\in R^{n}\left|c_{j}\left(x\right)\right.=0,c_{i}\left(x\right)\leq 0,j=1,\cdots ,m_{e},i=m_{e}+1,\cdots ,m\right\}, \end{array}$$

where *c*_*j*_(**x**) = 0 and *c*_*i*_(**x**) ≥ 0 are the equality and inequality constraints of multiobjective optimization, respectively. If *U* = *R*^*n*^, (1) is called an unconstrained multiobjective optimization problem.

For solving (1), we provide the concept of Pareto optimality as explained below.

**Definition 1 .**

A point *x*^∗^ ∈ *U* is a local Pareto optimum or local Pareto optimal solution of *f*(*x*) if and only if there does not exist *x* ∈ *U* such that

(3) $$\begin{array}{c} f\left(x\right)\leq f\left(x^{*}\right),\;x\neq x^{*}. \end{array}$$

Note that if *U* and *f*(*x*) are both convex, then the local Pareto optimality is equivalent to the global Pareto optimality. So a Pareto optimal solution means the reasonable solution, which satisfies the objectives at an acceptable level without being dominated by any other solution.

In order to obtain the information of every objective function and the change tendency of optimization process more intuitively, the set of objective function values can be used in case of making impolitic decision. The detailed definition is as below.

**Definition 2 .**

If *V* ⊂ *R*^*n*^ is the set of Pareto optimal solutions in (1), then set *P* is a Pareto front of *V* for which

(4) $$\begin{array}{c} P=\left\{F\left(x\right)=\left(f_{1}\left(x\right),f_{2}\left(x\right),\cdots ,f_{l}\left(x\right)\right)\left|x\in V\right.\right\} \end{array}$$

holds.

Assume *f*_*j*_(**x**) is twice continuously differentiable on feasible region *U*, i.e., *f*_*j*_(**x**) ∈ *C*^2^(*U*). And for **x** ∈ *U* ⊂ *R*^*n*^, let *∇f*_*j*_(**x**) ∈ *R*^*n*^ and *∇f*_*j*_^2^(**x**) ∈ *R*^*n*×*n*^ denote the gradient and Hessian matrix of *f*_*j*_(**x**) at **x** for all *j* = 1, 2,…, *m*, respectively.

Throughout the paper, unless explicitly mentioned, we will assume that *f*_*j*_(**x**) ∈ *C*^2^(*U*) with strong convexity which implies the *∇f*_*j*_^2^(**x**) is positive definite for all **x** ∈ *U* ⊂ *R*^*n*^, and *j* = 1, 2,…, *m*.

### 2.2. Newton Method Based on Weighted Sum Technique

Newton's method is extensively used in optimization problem, and the iteration direction includes the gradient and Hessian matrix information of objectives. When the initial iteration point is very close to the optimal point, the rate of convergence is rapid. And if the objective functions satisfy some conditions, it can achieve superlinear convergence or quadratic convergence. So in multiobjective optimization problem, Newton method combined with weighted sum method is chosen as the main calculation algorithm. The derivation process is as follows.

In the multiobjective optimization problem (1), *f*_*j*_(**x**) ∈ *C*^2^(*U*), the Taylor expansion of *f*_*j*_(**x**) around **x**_*k*_ ∈ *U* is

(5) $$\begin{array}{c} f_{j}\left(x\right)=f_{j}\left(x_{k}\right)+\nabla f_{j}\left(x_{k}\right)\left(x-x_{k}\right)+\frac{1}{2}{\left(x-x_{k}\right)}^{T}\nabla^{2}f_{j}\left(x_{k}\right)\left(x-x_{k}\right)+ο\left({\left\|x-x_{k}\right\|}^{2}\right). \end{array}$$

Hence, the second order approximate Taylor expansion of *f*_*j*_(**x**) around **x**_*k*_ ∈ *U* is

(6) $$\begin{array}{c} f_{j}\left(x\right)\approx f_{j}\left(x_{k}\right)+\nabla f_{j}\left(x_{k}\right)\left(x-x_{k}\right)+\frac{1}{2}{\left(x-x_{k}\right)}^{T}\nabla^{2}f_{j}\left(x_{k}\right)\left(x-x_{k}\right). \end{array}$$

Here, in (6), *∇f*_*j*_^2^(**x**_*k*_) is positive definite. Hence, the problem is converted from finding the minimum of *f*_*j*_(**x**) into finding the second order approximation minimum of *f*_*j*_(**x**). Since from the derivative of (6) at both sides with respect to **x**, using the necessary condition of extreme value, we obtain

(7) $$\begin{array}{c} \nabla f_{j}\left(x_{k}\right)+\nabla^{2}f_{j}\left(x_{k}\right)\left(x-x_{k}\right)=0. \end{array}$$

Considering the algorithm is an iterative process, take **x** = **x**_*k*+1_, we have the Newton iteration method for single objective as

(8) $$\begin{array}{c} x_{k+1}=x_{k}-{\left[\nabla^{2}f_{j}\left(x_{k}\right)\right]}^{-1}\nabla f_{j}\left(x_{k}\right). \end{array}$$

And note the iteration direction of Newton's method for a single objective at **x**_*k*_

(9) $$\begin{array}{c} d_{k}=x_{k+1}-x_{k}=-{\left[\nabla^{2}f_{j}\left(x_{k}\right)\right]}^{-1}\nabla f_{j}\left(x_{k}\right). \end{array}$$

For solving problem (1), by weighted sums of *f*_*j*_(**x**) for all *j* = 1, 2,…, *m*, we have the sum function which will be denoted by *F*_*λ*_(**x**). Hence,

(10) $$\begin{array}{c} F_{\lambda }\left(x\right)=\overset{l}{\underset{j=1}{\sum }}\lambda_{j}f_{j}\left(x\right)=\overset{l}{\underset{j=1}{\sum }}\lambda_{j}\left[f_{j}\left(x_{k}\right)+\nabla f_{j}{\left(x_{k}\right)}^{T}d_{k}+\frac{1}{2}{d_{k}}^{T}\nabla^{2}f_{j}\left(x_{k}\right)d_{k}\right], \end{array}$$

where the weighting factors will be denoted by *λ*_*j*_ and *λ*_*j*_ ≥ 0, ∑_*j*=1_^*l*^*λ*_*j*_ = 1 for all *j* = 1, 2,…, *l*.

Expression (10) can be calculated as the derivative at both sides with respect to **d**_*k*_, then, the iterative formula of Newton weighted sum algorithm for (1) is

(11) $$\begin{array}{c} x_{k+1}=x_{k}-{\left[\overset{l}{\underset{j=1}{\sum }}\lambda_{j}\nabla^{2}f_{j}\left(x_{k}\right)\right]}^{-1}\overset{l}{\underset{j=1}{\sum }}\lambda_{j}\nabla f_{j}\left(x_{k}\right). \end{array}$$

So, we can conclude the direction of Newton weighted sum algorithm for (1) at **x**_*k*_

(12) $$\begin{array}{c} d_{k}=-{\left[\overset{l}{\underset{j=1}{\sum }}\lambda_{j}\nabla^{2}f_{j}\left(x_{k}\right)\right]}^{-1}\overset{l}{\underset{j=1}{\sum }}\lambda_{j}\nabla f_{j}\left(x_{k}\right). \end{array}$$

## 3. Neumann Series Expansion

The Neumann series is the expansion of the matrix inversion, and its function lies in the efficiency of the matrix inversion. In engineering, the problem can be solved by the Newton method, and when there are many design variables, a considerable amount of calculation is needed. But when there are two object functions, the introduction of expansion principle can not only maintain the advantages of the Newton method but also reduce the work by half that is needed to run two object functions. The theorem is as follows:

**Theorem 1 .**

Assume that **K** ∈ *R*^*n*×*n*^ is a *n*-order invertible matrix. Then, for matrix Δ**K** ∈ *R*^*n*×*n*^, there exists **N** = **K**^−1^Δ**K**, and when ‖**N**‖ < 1, such

(13) $$\begin{array}{c} {\bar{K}}^{-1}={\left(K+\Delta K\right)}^{-1}={\left(K\left(I+K^{-1}\Delta K\right)\right)}^{-1}={\left(I+N\right)}^{-1}K^{-1}, \end{array}$$

(14) $$\begin{array}{c} {\bar{K}}^{-1}={\left(I-N+N^{2}+\cdots +{\left(-1\right)}^{m}N^{m}+\cdots \right)}^{-1}K^{-1} \end{array}$$

hold for *m* ∈ *N*_+_. (14) is called the Neumann series expansion.

According to (10), the weighted sum function of problem (31) is

(15) $$\begin{array}{c} F_{\lambda }\left(x\right)=\overset{2}{\underset{j=1}{\sum }}\lambda_{j}f_{j}\left(x\right), \end{array}$$

and the corresponding Newton's iterative format is

(16) $$\begin{array}{c} x_{k+1}=x_{k}-{\left[\overset{2}{\underset{j=1}{\sum }}\lambda_{j}\nabla^{2}f_{j}\left(x_{k}\right)\right]}^{-1}\overset{2}{\underset{j=1}{\sum }}\lambda_{j}\nabla f_{j}\left(x_{k}\right). \end{array}$$

Then the iterative direction vector of (16) at **x**_*k*_ is

(17) $$\begin{array}{c} d_{k}=-{\left[\overset{2}{\underset{j=1}{\sum }}\lambda_{j}\nabla^{2}f_{j}\left(x_{k}\right)\right]}^{-1}\overset{2}{\underset{j=1}{\sum }}\lambda_{j}\nabla f_{j}\left(x_{k}\right) \end{array}$$

According to (17) and Neumann series expansion of Theorem 1, let **K** be the square matrix *λ*_1_*∇*^2^*f*_1_(**x**_*k*_) in (13), and Δ**K** be the square matrix *λ*_2_*∇*^2^*f*_2_(**x**_*k*_) in (13), therefore,

(18) $$\begin{array}{c} N=K^{-1}\Delta K={\left(\lambda_{1}\nabla^{2}f_{1}\left(x_{k}\right)\right)}^{-1}\left(\lambda_{2}\nabla^{2}f_{2}\left(x_{k}\right)\right)=\frac{\lambda_{2}}{\lambda_{1}}{\left(\nabla^{2}f_{1}\left(x_{k}\right)\right)}^{-1}\nabla^{2}f_{2}\left(x_{k}\right). \end{array}$$

Define *λ* = *λ*_2_/*λ*_1_ > 0 with *λ*_1_ + *λ*_2_ = 1. Then we should state ‖**N**‖ < 1 holds with *λ* adjusted under some certain conditions.

Assume that *U* ⊂ *R*^*n*^ is a bounded set, for **x** = (*x*_1_, *x*_2_,…,*x*_*n*_)^*T*^ ∈ *U*, then *G* : *R*^*n*^⟶*R*^*n*×*n*^ defined by (19) is a linear operator.

(19) $$\begin{array}{c} G\left(x\right)=\nabla^{2}f_{j}\left(x\right)\in R^{n\times n}. \end{array}$$

Boundedness and continuity of *G*(**x**) are as follows.

**Theorem 2 .**

A linear operator *G* : *R*^*n*^⟶*R*^*n*×*n*^ is bounded if and only if there exists a constant *M* > 0, such that

(20) $$\begin{array}{c} \left\|Gx\right\|\leq M\left\|x\right\|,\;x\in U \end{array}$$

holds.

**Proof**

Assume that *G* is a bounded linear operator, so the unit ball $\bar{B_{1}}\left(\theta \right)=\left\{x\in R^{n}|\left\|x\right\|\leq 1\right\}$ can be mapped into a bounded set on *R*^*n*×*n*^ by *G*, i.e., the image set of $\bar{B_{1}}\left(\theta \right)$ is a bounded set on *R*^*n*×*n*^.

Take $M=\sup\left\{\left\|Gx\right\||x\in \bar{B_{1}}\left(\theta \right)\right\}$. If *θ* ∈ *U*, then *θ* satisfies to (22). For any **x** ∈ *U*, **x** ≠ *θ*, we have $x/\left\|x\right\|\in \bar{B_{1}}\left(\theta \right)$ and

(21) $$\begin{array}{c} \left\|G\left(\frac{x}{\left\|x\right\|}\right)\right\|\leq M, \end{array}$$

i.e.,

(22) $$\begin{array}{c} \left\|Gx\right\|\leq M\left\|x\right\|,\;x\in U,x\neq \theta . \end{array}$$

Therefore, if *G* is a bounded linear operator, then (22) holds.

Inversely, assume that (22) holds. The boundedness of *U* ⊂ *R*^*n*^ implies that there exists a positive constant *M*_1_ such that

(23) $$\begin{array}{c} \left\|a\right\|\leq M_{1},\text{for }a\in U. \end{array}$$

And there exists **a** ∈ *U*, such that

(24) $$\begin{array}{c} y=Ga \end{array}$$

holds for every **y** ∈ *GU*. From (20), we have

(25) $$\begin{array}{c} \left\|y\right\|=\left\|Ga\right\|\leq M\left\|a\right\|\leq M\cdot M_{1}. \end{array}$$

Therefore, sup{‖**y**‖: **y** = *G ***a**, **a** ∈ *U*} ≤ *M* · *M*_1_, i.e., *GU* is a bounded set on ℝ^*n*×*n*^.

**Theorem 3 .**

A linear operator *G* : *R*^*n*^⟶*R*^*n*×*n*^ is continues if and only if *G* is bounded.

**Proof**

(The necessary condition). Assume *G* is unbounded, then the inequality (20) is not satisfied. Hence, if there exists **x**_*n*_ ∈ *R*^*n*^, such that

(26) $$\begin{array}{c} \left\|Gx_{n}\right\|>n\left\|x_{n}\right\| \end{array}$$

holds for any natural number *n*. Take **y**_*n*_ = **x**_*n*_/*n*‖**x**_*n*_‖, we have

(27) $$\begin{array}{c} \left\|Gy_{n}\right\|>1,\left\|y_{n}\right\|=\frac{1}{n}⟶0\left(n⟶\infty \right), \end{array}$$

therefore, **y**_*n*_⟶*θ* but *G ***y**_*n*_⟶*θ* which is contradictious to the continuity of *G*.

(The sufficient condition). From the inequality (20), if **x**_*n*_⟶*θ*, then we have

(28) $$\begin{array}{c} \left\|Gx_{n}\right\|\leq M\left\|x_{n}\right\|⟶0\left(n⟶\infty \right), \end{array}$$

therefore, *G ***x**_*n*_⟶*θ* and *G* is continuous.

Because of *G*(**x**) = *∇*^2^*f*_*j*_(**x**) and under the assumption of *∇*^2^*f*_*j*_(**x**) is continuous matrix function for *j* = 1, 2. Therefore, the linear operator *G* is bounded on *U* ⊂ *R*^*n*^.

From the foregoing, we concluded that *G* is a continuous and bounded linear operator, so ‖*G*(**x**)‖ has an upper bound *W*_1_ on *U*. Similarly, linear operator *G*^−1^(**x**) = *∇*^2^*f*_*j*_(**x**)^−1^ and ‖*G*^−1^(**x**)‖ have their own upper bound *W*_1_′ = 1/*W*_1_ on *U* for *j* = 1, 2. So, back to (18) and the compatibility of norms, we can have

(29) $$\begin{array}{c} \left\|N\right\|=\frac{\lambda_{2}}{\lambda_{1}}\left\|{\left(\nabla^{2}f_{1}\left(x_{k}\right)\right)}^{-1}\nabla^{2}f_{2}\left(x_{k}\right)\right\|\leq \frac{\lambda_{2}}{\lambda_{1}}\left\|\nabla^{2}f_{1}{\left(x_{k}\right)}^{-1}\right\|\cdot \left\|\nabla^{2}f_{2}\left(x_{k}\right)\right\|. \end{array}$$

In (29), ‖*∇*^2^*f*_1_(**x**_*k*_)^−1^‖ · ‖*∇*^2^*f*_2_(**x**_*k*_)‖ has a public upper bound which denoted by *W* > 0. Hence, it can fully satisfy the requirement of ‖**N**‖ < 1 by adjusting *λ* = *λ*_2_/*λ*_1_ appropriately.

## 4. An Algorithm for Unconstrained Biobjective Optimization Problem Based on Neumann Series Expansion (NSE)

When there are only two objective functions, a biobjective optimization algorithm is established in this paper by introducing the technique of NSE [21] to Newton weighted sum method. With this algorithm, the complicated inverse calculation of *n*-rank matrix is avoided. It is only needed to calculate the inversion once for Hessian matrix of one objective function in (1). So the operating speed is improved especially in a high-dimensional design variables condition. Hence, the proposed algorithm is named as Newton Neumann Series Expansion Algorithm (NNSEA).

When there are only two objective functions, rewrite the problem (1) as

(30) $$\begin{array}{c} \begin{array}{c} F\left(x\right)=\min\;{\left(f_{1}\left(x\right),f_{2}\left(x\right)\right)}^{T},\\ s.t.\;\left\{\begin{array}{c} c_{i}\left(x\right)=0,\;i=1,2,\ldots ,m_{e},\\ c_{i}\left(x\right)\leq 0,\;i=m_{e}+1,\ldots ,m. \end{array}\right. \end{array} \end{array}$$

Note that when the feasible region *U* is extended to ℝ^*n*^, the constraints are invalid and (30) can be turned into unconstrained biobjective optimization as

(31) $$\begin{array}{c} \underset{x\in {\mathbb{R}}^{n}}{F\left(x\right)}=\underset{x\in {\mathbb{R}}^{n}}{\min}{\left(f_{1}\left(x\right),f_{2}\left(x\right)\right)}^{T}. \end{array}$$

The whole process of NNSEA for calculating a biobjective Pareto optimal solution is symbolized by Algorithm 1 as follows.

**Figure 1:**
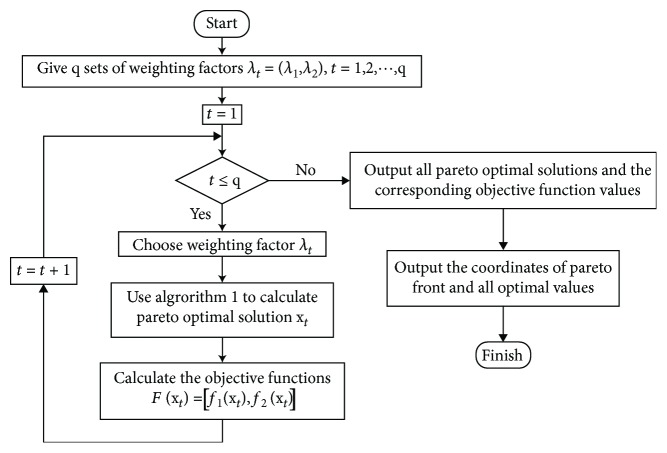
The process of NNSEA for solving unconstrained biobjective optimization.

By selecting multiple groups of weighting factors, each group can obtain a Pareto optimal solution and the corresponding Pareto front. The process of NNSEA for solving unconstrained biobjective is shown in Figure 1.

## 5. NNSEFA Based on NNSEA

NNSEA is a method for solving unconstrained biobjective optimization problem. But the design variables are mostly under some inequality constraints in engineering application. Therefore, to improve the NNSEA by handling constraints is of great significance in engineering case. By introducing a penalty function, the constraints can be transformed to penalty terms and integrated into objective functions. Based on the NNSEA and combined with Frisch's penalty function method, the proposed algorithm for solving constrained biobjective optimization is named Newton Neumann Series Expansion Frisch Algorithm (NNSEFA).

### 5.1. Handling the Constraints with Frisch Penalty Function

The Frisch penalty function is one of the interior penalty function methods by employing a logarithm to handle with the constraint. In problem (30) with constraints

(32) $$\begin{array}{c} c_{i}\left(x\right)\leq 0,\;i=1,2,\ldots ,m. \end{array}$$

The penalty term is constructed by Frisch's method, which is expressed as

(33) $$\begin{array}{c} q\left(x\right)=-\frac{1}{\sigma }\overset{m}{\underset{i=1}{\sum }}\log\left(-c_{i}\left(x\right)\right). \end{array}$$

When the penalty term *q*(**x**) is closer to zero, it means that design variables satisfy the constraints. During the solving process, *q*(**x**) should be scaled down until it is small enough to be neglected compared to the object values. At this moment, the obtained solution can not only be equivalent to the optimal solution of the original problem but also satisfy the constraints. The process of NNESFA is as follows.

First, denote the logarithmic penalty function of *f*_*j*_(**x**) as

(34) $$\begin{array}{c} p_{j}\left(x\right)=f_{j}\left(x\right)-\frac{1}{\sigma }\overset{m}{\underset{i=1}{\sum }}\log\left(-c_{i}\left(x\right)\right), \end{array}$$

the Taylor expansion of *p*_*j*_(**x**) around **x**_*k*_ ∈ *U* is

(35) $$\begin{array}{c} p_{j}\left(x\right)=p_{j}\left(x_{k}\right)+\nabla p_{j}\left(x_{k}\right)\left(x-x_{k}\right)+\frac{1}{2}{\left(x-x_{k}\right)}^{T}\nabla^{2}p_{j}\left(x_{k}\right)\left(x-x_{k}\right)+ο\left({\left\|x-x_{k}\right\|}^{2}\right). \end{array}$$

Hence, the second order approximation Taylor expansion of *p*_*j*_(**x**) around **x**_*k*_ ∈ *U* is

(36) $$\begin{array}{c} p_{j}\left(x\right)\approx p_{j}\left(x_{k}\right)+\nabla p_{j}\left(x_{k}\right)\left(x-x_{k}\right)+\frac{1}{2}{\left(x-x_{k}\right)}^{T}\nabla^{2}p_{j}\left(x_{k}\right)\left(x-x_{k}\right). \end{array}$$

Then, by adding *p*_*j*_(**x**) together for *j* = 1, 2, a sum function denoted by *P*_*λ*_(**x**) which can be expressed as

(37) $$\begin{array}{c} P_{\lambda }\left(x\right)=\overset{2}{\underset{j=1}{\sum }}\lambda_{j}p_{j}\left(x\right)=\overset{2}{\underset{j=1}{\sum }}\lambda_{j}\left[p_{j}\left(x_{k}\right)+\nabla p_{j}{\left(x_{k}\right)}^{T}d+\frac{1}{2}d^{T}\nabla^{2}p_{j}\left(x_{k}\right)d\right], \end{array}$$

where ∑_*j*=1_^2^*λ*_*j*_ = 1 and *λ*_*j*_ > 0.

By taking derivative of (37) at both sides with respect to **d**, the iterative formula is

(38) $$\begin{array}{c} x_{k+1}=x_{k}-{\left[\overset{2}{\underset{j=1}{\sum }}\lambda_{j}\nabla^{2}p_{j}\left(x_{k}\right)\right]}^{-1}\overset{2}{\underset{j=1}{\sum }}\lambda_{j}\nabla p_{j}\left(x_{k}\right). \end{array}$$

So, the iteration direction at **x**_*k*_ is

(39) $$\begin{array}{c} d_{k}=-{\left[\overset{2}{\underset{j=1}{\sum }}\lambda_{j}\nabla^{2}p_{j}\left(x_{k}\right)\right]}^{-1}\overset{2}{\underset{j=1}{\sum }}\lambda_{j}\nabla p_{j}\left(x_{k}\right). \end{array}$$

Just like NNSEA, by calculating **N** = *λ*_2_/*λ*_1_[*∇*^2^*p*_1_(**x**_*k*_)]^−1^*∇*^2^*p*_2_(**x**_*k*_), and based on the valve of ‖**N**‖, we choose the direction properly. In order to ensure all the directions are descending during the optimization, an identification process is introduced. Taking the negative gradient direction of sum function, i.e., **d**_*k*_ = −∑_*j*=1_^2^*λ*_*j*_*∇p*_*j*_(**x**_*k*_). The criterion of this strategy is the product of Newton direction and negative gradient direction denoted by

(40) $$\begin{array}{c} a_{k}=-{\left(\overset{2}{\underset{j=1}{\sum }}\lambda_{j}\nabla p_{j}\left(x_{k}\right)\right)}^{T}{\left(\overset{2}{\underset{j=1}{\sum }}\lambda_{j}\nabla^{2}p_{j}\left(x_{k}\right)\right)}^{-1}\overset{2}{\underset{j=1}{\sum }}\lambda_{j}\nabla p_{j}\left(x_{k}\right). \end{array}$$

### 5.2. NNSEFA for Constrained Biobjective Optimization Problem

The whole process of NNSEFA for calculating a constrained biobjective Pareto optimal solution is symbolized by Algorithm 2 as follows.

**Figure 2:**
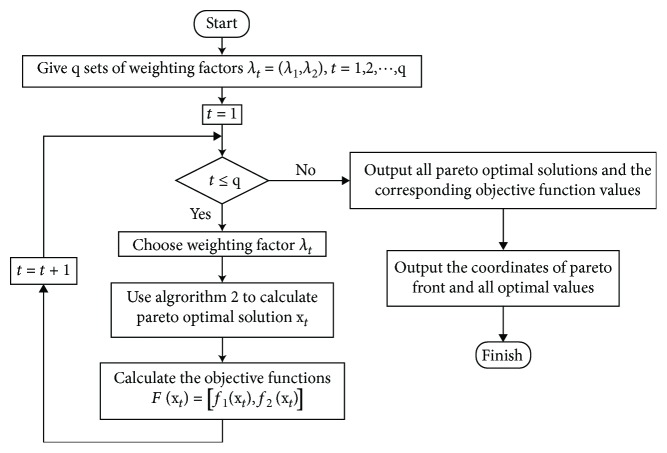
The process of NNSEFA for solving inequality constraints biobjective optimization.

Similarly, by selecting multiple groups of weighting factors, each group can obtain a Pareto optimal solution and the corresponding Pareto front. The process of NNSEFA for solving inequality constrained biobjective is shown in Figure 2.

## 6. Numerical Examples

Two benchmark test examples are chosen to test the effectiveness of NNSEA and NNSEFA. The first is an example from a published paper [22] for NNSEA. The second one is from the Genetic Algorithm Toolkit (GATOOL) of MATLAB for NNSEFA.

### 6.1. Test 1

(41) $$\begin{array}{c} \min F\left(x\right)=\left\{\begin{array}{c} F_{1}={\left(x_{1}-1\right)}^{2}+{\left(x_{1}-x_{2}\right)}^{2},\\ F_{2}={\left(x_{2}-3\right)}^{2}+{\left(x_{1}-x_{2}\right)}^{2}. \end{array}\right. \end{array}$$

**Figure 3:**
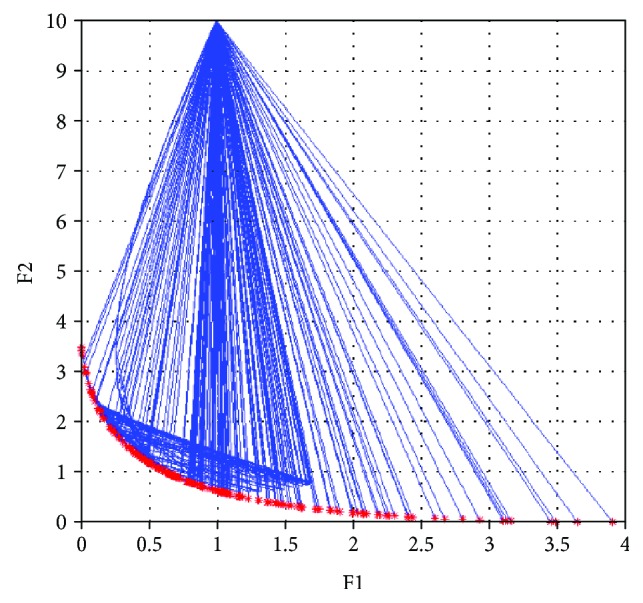
The Pareto optimal front of test 1 (random distribution).

**Figure 4:**
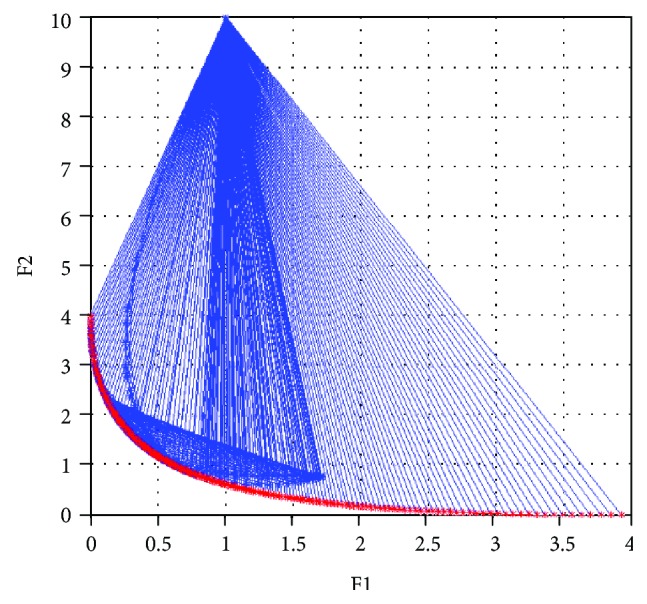
The Pareto optimal front of test 1 (uniform distribution).

Start coding in MATLAB 7.9.0(R2009b) for NNSEA, and set *ε* = 10^−5^, weighting factors **λ** = (*λ*_1_, *λ*_2_) are generated in (0, 1). Initial point **x**_0_ = (*x*_1_, *x*_2_)^*T*^ = (1, 0)^*T*^. Note that the weighting factors are generated in two approaches, one is generated randomly, and another is provided uniformly. Then start the calculation and output the coordinates of Pareto front with all 200 groups of optimal solutions in Figures 3 and 4.

As can be seen in Figures 3 and 4, each red asterisks represents a Pareto optimal function value of one specific set of weighting factor. And all the function values compose the Pareto front. The blue asterisks and blue lines represent the iterative points and descent directions in the whole process. 200 groups of Pareto optimal solutions are all convergent and distributed uniformly and broadly. In this paper, the calculations are executed 20 times by each weighting factor generate approach, the detailed information is listed in Table 1. It is clear that this NNSEA has high performance and good adaptability for biobjective optimization problem.

### 6.2. Test 2

(42) $$\begin{array}{c} \begin{array}{c} \min\;F\left(x\right)=\left\{\begin{array}{c} F_{1}=x_{1}^{4}-10x_{1}^{2}+x_{1}x_{2}+x_{2}^{4}-x_{1}^{2}x_{2}^{2},\\ F_{2}=x_{2}^{4}-x_{1}^{2}x_{2}^{2}+x_{1}^{4}+x_{1}x_{2}, \end{array}\right.\\ s.t.\;\left\{\begin{array}{c} -5\leq x_{1}\leq 5,\\ -5\leq x_{2}\leq 5. \end{array}\right. \end{array} \end{array}$$

**Figure 5:**
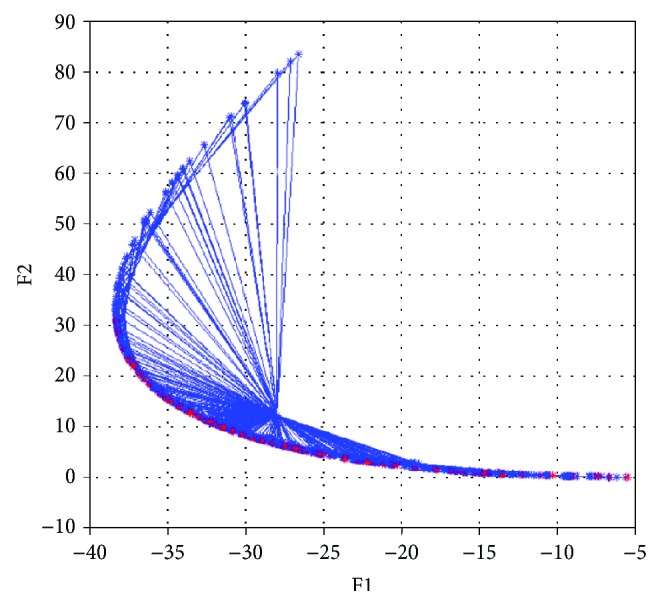
The Pareto optimal front of test 2 (contains each iteration).

Similarly, in test 2 we set *ε* = 10^−5^ and weighting factors are generated in (0, 1) randomly. Initial point is **x**_0_ = (*x*_1_, *x*_2_)^*T*^ = (2,−2)^*T*^, and set coefficients *α* = 0.9, *ρ* = 0.9, *η* = 0.05 with initial *σ* = 2, the iterative number for calculating every Pareto optimal solution is no more than 200. Then start the calculation and output the coordinates of Pareto front with all 200 groups of optimal solutions in Figure 5.

**Figure 6:**
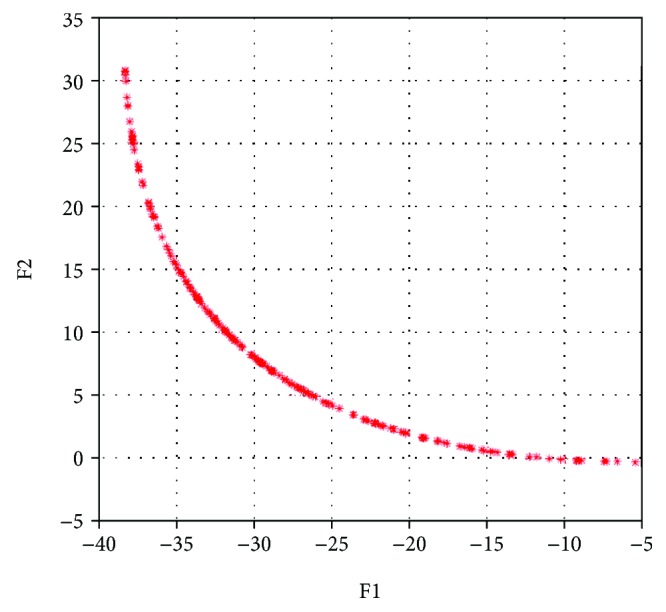
The Pareto optimal front of test 2.

Only the Pareto front is displayed for giving an observation of function values more clearly. In the biobjective optimization problem, the middle section of Pareto front should be emphatically focused because of the values of this section, without favoring either function. We can see the middle section of the red asterisks, which are arranged smoothly and compactly, in Figure 6. Executed 20 times in the same computer and recorded, the average time of calculation is 270.85 s with the average number of iterative for each Pareto solution of 8.54, and all the Pareto optimal solutions are convergent which means NNSEFA has a good performance of efficiency and convergence.

## 7. Biobjective Optimization for Dummy Head in Car Crash Experiment

With the rapid development of automobile industry, the passive safety of automobiles has become a more and more important research subject for enterprises and research institutes. As an anthropomorphic test device, collision dummies are widely used in automobile safety testing. The collision dummy is made up of the head, neck, chest, buttocks, upper limbs, and lower limbs. Head injury is one of the most common injuries in traffic accidents, and statistics show that the mortality caused by head injury is the highest, which accounts for 68% of all deaths, making head injury the greatest killer in car accidents [23]. Therefore, research on dummy head structure in automobile crashes is essential to head injury analysis and vehicle safety.

### 7.1. Modeling of Biobjective Optimization Problem for Dummy Head Design

**Figure 7:**
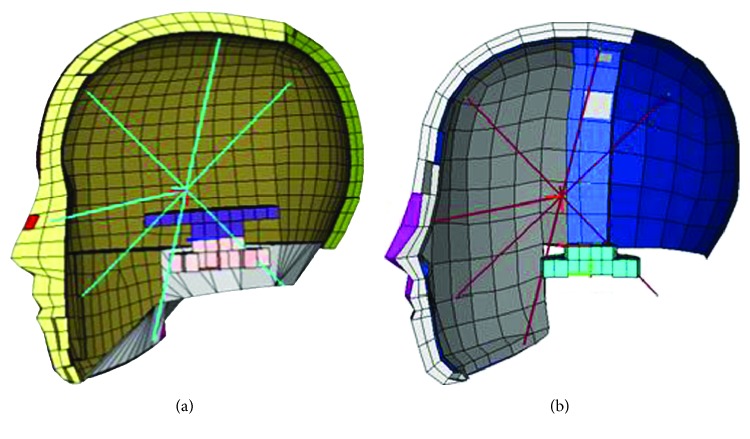
Detailed FE models of Hybrid III 50th and the simplified head structure.

In this paper, to improve the efficiency of the dummy head analysis, a simplified dummy head model is developed based on the finite element (FE) model of Hybrid III 50^th^. Sensitivity analysis and the equivalent modeling in mechanics are applied to developing the new simplified dummy head structure. Through sensitivity analysis of the materials used for dummy head structure such as artificial skin and bones and mechanical property response, a simplified dummy head model is proposed in this section. In Figure 7(b), the detailed FE model of the proposed simplified head model is given and the dummy head FE model of Hybrid III 50th is shown in Figure 7(a). All models in this paper are meshed by software HyperMesh and then solved in LS-DYNA and meshed with shell and body elements.

**Figure 8:**
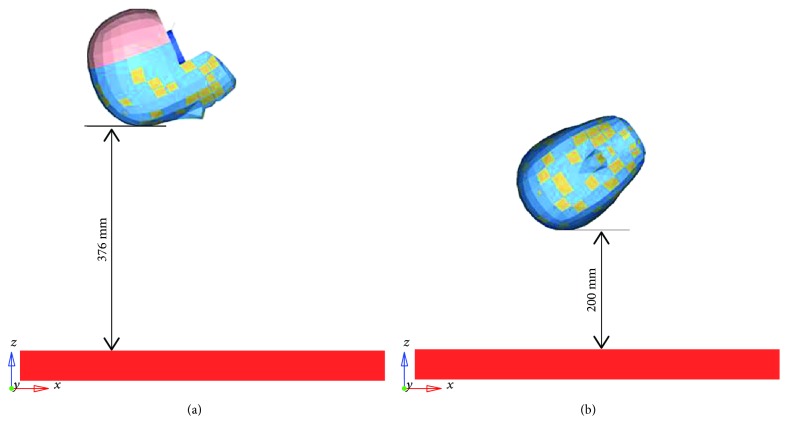
Two forms of frontal and lateral impacts.

For the dummy head calibration test, there is a legal and authoritative standard to examine the validity of the dummy structure. The synthetic head acceleration generated in automobile crashes is applied to evaluating the mechanical properties of the head structure. Considering the frontal and lateral impact condition, the peak accelerations of the dummy head generated in frontal and lateral collision are denoted as *a*_frb_ and *a*_mdb_, respectively. The accuracy of the dummy head model is comprehensively considered by using the method of frontal and lateral head regulation, and thus the peak *a*_frb_ and *a*_mdb_ are used as the main indexes of the mechanical characteristics of the dummy head. In simulating the collision process of the head structure, the models drop from a certain height and hit a rigid plate, as shown in Figure 8. The simulation processes of the dummy head in frontal and lateral collisions are presented in Figures 8(a) and 8(b), respectively.

### 7.2. Validity of the Dummy Head Model

**Figure 9:**
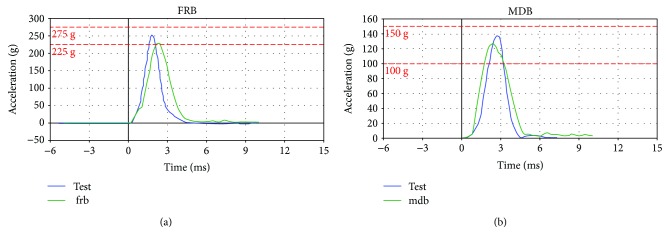
The acceleration-time curves of the dummy head model under frontal and lateral collisions.

Acceleration curves of the simplified dummy head models under frontal (FRB) and lateral (MDB) impacts are obtained, and calibration test is referred for comparison analysis. Thus, two acceleration-time curves compared with the test curves of the dummy head model under different collision stations are depicted in Figure 9. In Figure 9, the green curves are obtained in simulated dummy test, and the blue curves in physical dummy test. Moreover, Figure 9(a) is in frontal impact and Figure 9(b) in lateral collision.

According to Figure 9, the acceleration-time curves of the simplified dummy head model proposed in this paper is consistent with the experimental results, and also, the two peak acceleration values *a*_frb_ and *a*_mdb_ meet the calibration requirement. The quantitative analysis of the two peak accelerations compared with the test results are carried out in Table 2, and the errors in peak acceleration between the simplified model and physical dummy head are also considered in this paper.

From Table 2, we can conclude that the two peak acceleration values are all within calibration range, which means that the simplified dummy head model developed in this paper is effective and can be used in the simulation research of the head injuries. However, the figures in Table 2 also present a fact that the error of the peak acceleration of simplified head model is greater; thus, the accuracy of the head model cannot be guaranteed in some cases. Therefore, an optimization design of the dummy head structure will be carried out in the following section to improve the accuracy of the simplified head model.

### 7.3. Establishment of Mathematical Model for Optimization Design of the Dummy Head

According to Wood et al. [24], the mechanical responses of dummy head in automobile collision are typically dependent on the viscoelastic properties of the head polymer skin. In order to improve the accuracy of the simplified dummy head proposed in this paper, three material parameters—relaxation modulus (GI), shear modulus (MU1), and decay constant (BETA)—are selected as design variables based on the material sensitivity and the research on physical properties. Considering the frontal and lateral collision, thus the optimization design of the dummy head is a biobjective optimization problem. Thus, a biobjective optimization problems for optimization design of the dummy head structure is established as follows:

(43) $$\begin{array}{c} \left\{\begin{array}{c} \min\;\left(F_{1}=\left|a_{fr0}-a_{frb}\left(x\right)\right|,F_{2}=\left|a_{m0}-a_{mdb}\left(x\right)\right|\right),\\ s.t.\;l_{i}\leq x_{i}\leq u_{i},\;i=1,2,3, \end{array}\right. \end{array}$$

where *F*_1_ and *F*_2_ are two objectives; *a*_fr0_ = 250.14 g, and *a*_*m*0_ = 137.1 g are the experiment values of the peak acceleration of the dummy head, while *a*_frb_(*x*) and *a*_mdb_(*x*) are acceleration peak values of the simplified model under frontal and lateral impact conditions. And *x*_*i*_, *i* = 1, 2, 3 are design variables that consist of GI, MU1, and BETA; according to the calibration test design requirements, in this paper, *x*_1_ ∈ [0.0005, 0.005], *x*_2_ ∈ [7*e* − 4, 1*e* − 3], *x*_3_ ∈ [0.3, 1.2].

### 7.4. Establishment of Surrogate Model of Dummy Head

To reduce the complexity of the optimization problem in practical engineering, surrogate modeling has been broadly applied to many fields for its simple theory and excellent function. In this simulation, the response surface methodology (RSM) is selected for its better performance in many multiobjective optimization problems [25–27]. Firstly, 16 groups data (3 factors 4 levels) are obtained by the orthogonal experimental design (DOE) method, and the corresponding acceleration results performed in LS-DYNA are shown in Table 3.

According to the data in Table 3, the quadratic polynomial response surface models of the objective functions *F*_1_ and *F*_2_ are constructed as equation (44) and (45).

(44) $$\begin{array}{c} F_{1}=49.30481-16344.7x_{1}-2419.06x_{2}-38.0611x_{3}-101547x_{1}^{2}-3.9E+07x_{2}^{2}+4.4533029x_{3}^{2}+15829917x_{1}x_{2}+3693.5245x_{1}x_{3}+48179.205x_{2}x_{3}, \end{array}$$

(45) $$\begin{array}{c} F_{2}=26.32685-1454.22x_{1}-43582.2x_{2}+15.05433x_{3}+109443.5x_{1}^{2}+32371025x_{2}^{2}-0.72198x_{3}^{2}-2137901x_{1}x_{2}+1413.658x_{1}x_{3}-16641x_{2}x_{3}. \end{array}$$

Then, after surrogate models are obtained, the determination coefficient of variance analysis is employed to verify the fitting precision of response surface models. In general, if the determination coefficient is closer to 1, the surrogate model function with respect to response variables is more precise. In the paper, the determination coefficients *R*_frb_^2^ and *R*_mdb_^2^ [27] are 93.153% and 98.8691%, respectively. Therefore, the surrogate models in this paper can simulate response variables accurately.

### 7.5. Optimization Design of the Dummy Head Structure

**Figure 10:**
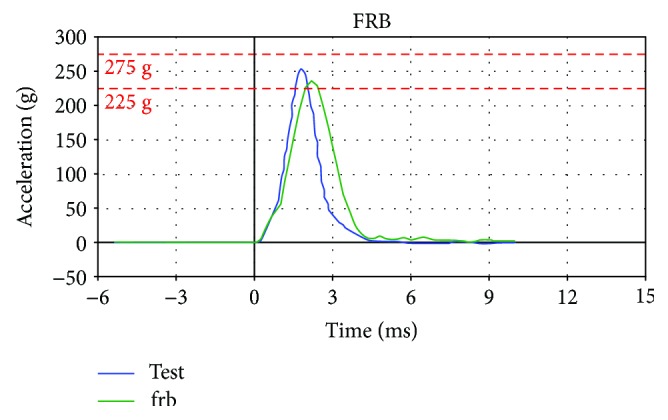
Acceleration-time curves of the dummy head under frontal collision.

**Figure 11:**
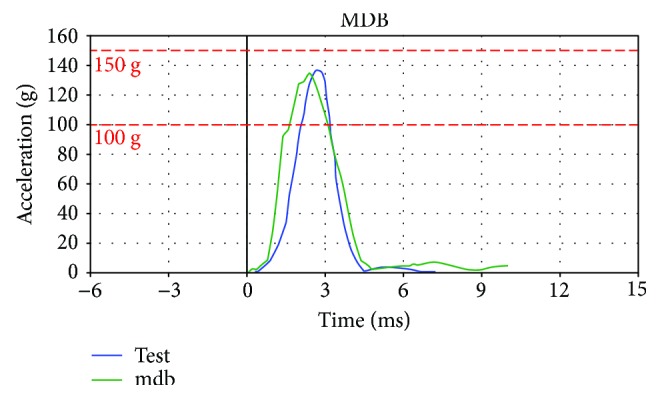
Acceleration-time curves of the dummy head under lateral collision.

The effective optimization strategy proposed in this paper is applied to the biobjective optimization design of the dummy head structure. Following the optimal process of the strategy NNSEFA, the Pareto optimal solutions of the biobjective problem (43) are obtained quickly and after comparative analysis, an optimal scheme with design variables *x*_1_ = 0.0056 Gpa, *x*_2_ = 6e-4 Gpa, and *x*_3_ = 0.3 is selected for optimization design of the simplified dummy head model. The two accelerations were considered to evaluate the performance of the optimal design. Figures 10 and 11 show the acceleration-time curves of the optimal dummy head model compared with the physical dummy head structure under frontal and lateral collision, respectively. Moreover, the green curves in Figures 10 and 11 are obtained through the simulated dummy head experiment and the blue curves through physical dummy test.

According to the acceleration-time curves presented in Figures 10 and, the optimal design of the dummy head structure satisfy the calibration requirement, and its peak value of the two accelerations is close to the acceleration value obtained in the experiment of the physical dummy head. In addition, the acceleration-time curves of the optimal design are consistent with the experimental results. To analyze the properties of the optimal dummy head model, the design scheme and its two corresponding responses are shown in Table 4.

From Table 4, the degree of the optimizations for the simplified dummy head model is obvious and appreciable. Firstly, the peak values of acceleration for the frontal and lateral drop of the dummy head model is in accordance with the calibration test completely. The peak synthesis acceleration of frontal head drop is 236.7 g, the error of the simulated model being −5.37%, reduced by 2.48% compared with the original model before optimization, and the synthetic acceleration peak in lateral drop is 136.0 g, the deviation from the peak of the test curve being −0.80%, reduced by 5.98% after optimization. It can be seen that the optimal model of the dummy head is not only effective but also accurate in calculation. In addition, the dummy head model has been simplified for high computation efficiency, and the biobjective optimization performed in the optimal design has made the model more accurate. In summary, the optimization scheme for dummy head structure is of high accuracy and can be applied to the study on the occupant safety protection.

## 8. Conclusion

Two algorithms*—*NNSEA and NNSEFA—are presented in this paper. In NNSEA, the Neumann series expansion is introduced in Newton's weighted sum method so it can simplify the calculation on matrix inversion. And based on NNSEA, Frisch penalty function is employed to handle the constraints. So NNSEFA can achieve biobjective optimization with constraints. When the objective functions are strong convex, the proposed NNSEA and NNSEFA are two efficient approaches for biobjective optimization problems according to the test function analysis.

In the engineering application of dummy, NNSEFA provides valuable reference to designers. As computer simulation shows, the general calibration curve, the peak and the peak time for the optimized model of dummy head, and physical dummy are very consistent. Compared with the original design, this optimization model can simulate the injury of human head in the collision process more accurately. It provides the design idea for occupant safety design. Obviously, the optimization is feasible.

## Figures and Tables

**Algorithm 1:**
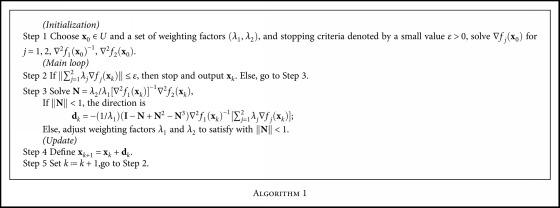


**Algorithm 2:**
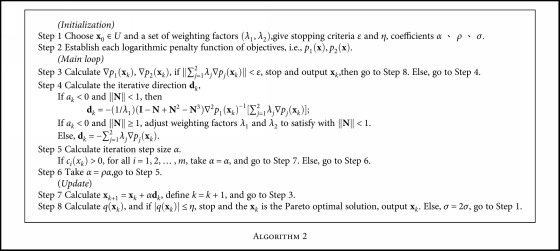


**Table 1:** The average calculating time and iteration information of NNSEA for solving test 1.

Weighting factor	Average time	Average number of iterations	Convergence
Random distribution	185.32 s	13.48	Yes
Uniform distribution	144.24 s	12.33	Yes

**Table 2:** Comparison of two peak accelerations between simplified head model and physical dummy head.

Model	Design variables			Acceleration	
	*x*_1_ (Gpa)	*x*_2_ (Gpa)	*x*_3_	*a*_frb_/g	*a*_mdb_/g
Original design	0.0015	7.5e-4	0.3	230.5	127.8
Calibration requirement	—	—	—	225–275	100–150
Test result	—	—	—	250.14	137.1
Error	—	—	—	−7.85%	−6.78%

**Table 3:** 16 groups obtained by DOE and its corresponding response.

Number	*x*_1_(GI)	*x*_2_(MU1)	*x*_3_(BETA)	|*a*_fr0_ − *a*_frb_|	|*a*_*m*0_ − *a*_mdb_|
1	0.0005	7e-4	0.3	25.64	11.2
2	0.0005	7.5e-4	0.6	24.54	11.8
3	0.0005	8e-4	0.9	25.24	12.1
4	0.0005	1e-3	1.2	26.64	11.1
5	0.0015	7e-4	0.6	23.24	10.9
6	0.0015	7.5e-4	0.3	19.64	9.3
7	0.0015	8e-4	1.2	30.24	11.8
8	0.0015	1e-3	0.9	27.84	10.0
9	0.0035	7e-4	0.9	19.44	9.4
10	0.0035	7.5e-4	1.2	29.24	9.9
11	0.0035	8e-4	0.3	11.84	4.0
12	0.0035	1e-3	0.6	16.94	5.7
13	0.005	7e-4	1.2	22.84	11.6
14	0.005	7.5e-4	0.9	17.44	7.0
15	0.005	8e-4	0.6	16.14	4.6
16	0.005	1e-3	0.3	12.34	1.4

**Table 4:** Detailed design variables and its corresponding responses compared with the original design and the result obtained by test.

Model	Design variables			Acceleration	
	*x*_1_ *x*_2_ *x*_3_ (Gpa) (Gpa)			*a*_frb_/g	*a*_mdb_/g
Original model	0.0015	7.5e-4	0.3	230.5	127.8
Optimal design	0.0054	6e-4	0.3	236.7	136.0
Calibration requirement	—	—	—	225–275	100–150 *reference goes here*
Test result	—	—	—	250.14	137.1
Error	—	—	—	−5.37%	−0.80%
Optimal percentage	—	—	—	2.48%	5.98%

## Data Availability

The data used to support the findings of this study are available from the corresponding author upon request.
